# NMR Studies of the Ion Channel-Forming Human Amyloid-β with Zinc Ion Concentrations

**DOI:** 10.3390/membranes11110799

**Published:** 2021-10-20

**Authors:** Minseon Kim, Jinyoung Son, Yongae Kim

**Affiliations:** Department of Chemistry, Hankuk University of Foreign Studies, Yongin 17035, Korea; alstjs032@naver.com (M.K.); jinyung7720@naver.com (J.S.)

**Keywords:** amyloid channel, transmembrane protein, Alzheimer’s disease, solution-state NMR, solid-state NMR

## Abstract

Alzheimer’s disease (AD) is classified as an amyloid-related disease. Amyloid beta (Aβ) is a transmembrane protein known to play a major role in the pathogenesis of AD. These Aβ proteins can form ion channels or pores in the cell membrane. Studies have elucidated the structure of the transmembrane domain of Aβ ion channels. In addition, various studies have investigated substances that block or inhibit the formation of Aβ ion channels. Zinc ions are considered as potential inhibitors of AD. In this study, we focused on the transmembrane domain and some external domains of the Aβ protein (hAPP-TM), and solution-state NMR was used to confirm the effect on residues of the protein in the presence of zinc ions. In addition, we sought to confirm the structure and orientation of the protein in the presence of the bicelle using solid-state NMR.

## 1. Introduction

Neurodegenerative diseases are incurable and debilitating conditions that result in progressive degeneration and nerve cell death. Parkinson’s and Huntington’s diseases are associated with a decline in mental ability accompanied by chorea or movement disability and both the neurodegenerative diseases are associated with aggregation and misfolding of amyloidogenic peptides [1,2,3,4]. Alzheimer’s disease (AD) is a well-known neurodegenerative disease that induces dementia via neuronal loss. Amyloid beta (Aβ) and metal ions are major pathological causative factors. Aβ is a protein fragment produced via proteolytic cleavage of the amyloid precursor protein (APP) by γ-secretases. Aβ is an integral transmembrane protein that plays a central role in the progression of AD [5,6,7,8].

The hypothesis of ion channel formation in AD states that the formation of an ion channel in the nerve cell membrane induced by the accumulated Aβ perturbs calcium ion homeostasis [9,10,11]. As a result, ions freely pass through the cell membrane, and the concentration of ions inside the cell changes, causing neuronal dysfunction, and apoptosis, leading to memory impairment [12,13].

Aβ ion channel or pore formation acts as a Ca^2+^-selective channel responsible for unregulated Ca^2+^ flux in the cytoplasm of brain cells [13,14,15,16]. The ability to induce ion flux suggests cytotoxicity [17,18,19,20]. In particular, Aβ(1–42) is known to contain a unique structural feature that promotes membrane passage and channel formation compared to Aβ(1–40) [16].The superficial structure and membrane potential of Aβ ion channel were studied via atomic force microscopy (AFM) and electrophysiological techniques to measure ion flux [16,17,21,22,23,24,25,26,27,28].

Various drugs are used to alleviate AD symptoms and delay disease progression. Several studies are currently investigating substances that block or inhibit the formation of Aβ ion channels. Among them, zinc ions are the most common transition metals regulating the signals emitted by glutamate neurons. Large concentrations of zinc ions in synaptic clefts are potential therapeutic or alleviating agents in AD [29]. Zinc ions bind with strong affinity to the Aβ ion channels, blocking one side of the ion channels and reducing their conductivity [13,24]. Since a decrease in conductivity implies a decrease in the flow of calcium ions, the possibility of restoring calcium ion homeostasis in nerve cells can be confirmed. Both Aβ40 and Aβ42 contain a hydrophilic N-terminus that contains a metal ion ligand [30,31]. We focused on the amino acid residues 692 and 723 (hAPP-TM), which have affinity for metal ions, including zinc ions, among the APPs [31].

In this study, we focused on the formation of the Aβ ion channel and demonstrated the ability of zinc ions to close the ion channel of the Aβ protein via structural changes in membrane proteins. We expressed a protein in *E. coli* with an amino acid sequence containing residues 692-723 of the transmembrane region of human APP (hAPP-TM) and a protein containing 8 residues of periplasmic domain using recombination techniques. The final purified protein was obtained using an optimized purification method, and the structure of hAPP-TM was investigated using various analytical methods. The protein structure in a biomembrane analog formed using phospholipids was investigated using solution-state and solid-state NMR. For accurate structural analysis, MATLAB was used based on the polarity index slant angle (PISA) wheel pattern and the mechanism of hAPP-TM protein was elucidated.

## 2. Materials and Methods

### 2.1. Expression and Purification of hAPP-TM

**Figure 1:**
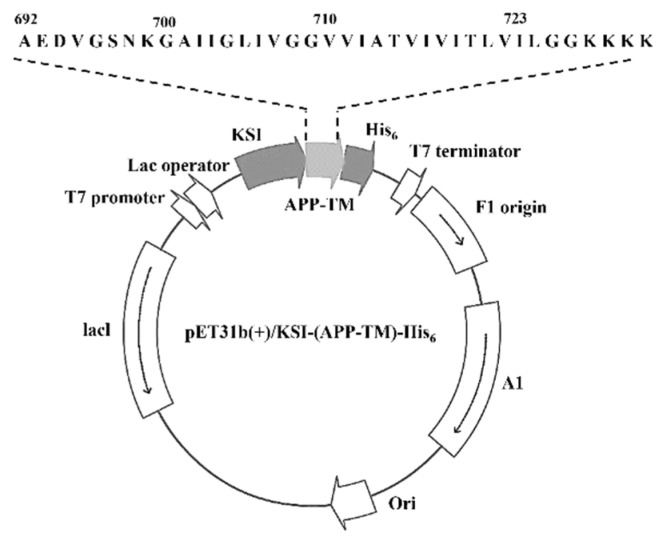
A schematic diagram of the expressed KSI-(APP-TM)-His_6_ DNA construct.

The 117-base oligonucleotide coding sequence for hAPP-TM was chemically synthesized by Integrated DNA Technologies (Coralville, IA, USA). The hAPP-TM consists of 38 amino acids containing ~6 moderately polar residues for improving solubility following ~24 apolar residues (GAIIGLIVGVVIATVIVITLVIL). *E. coli* C43(DE3) cells were transformed with the purified and inserted DNA containing the KSI (ketosteroid isomerase)-hAPP-TM-His_6_ into a pET31b (+) vector (Novagen Inc., Madison, WI, USA) (Figure 1). Detailed information on this has been described in a previous paper [32].

For the expression of the fusion protein, a starter culture was prepared by inoculating the plate containing LB medium supplemented with carbenicillin (Amresco, Solon, OH, USA) and the plates were incubated overnight at 37 °C in a shaker. A 10-mL aliquot of the fully grown culture was then transferred into 1 L of M9 minimal medium and the culture was grown at 37 °C. Uniformly ^15^N-enriched ammonium sulfate (Cambridge Isotope Lab., Tewksbury, MA, USA) was used for NMR structural analysis. When the optical density at wavelength 600 nm (OD_600_) reached 0.5~0.6, the fusion protein was induced via addition of 1 mM IPTG and cells were grown for an additional 14 h at 37 °C. After overnight incubation, the cells were harvested via pellet centrifugation (14,500 rpm, 4 °C, 30 min) and stored at −80 °C.

The frozen cell pellet from 1 L culture was resuspended in 100 mL lysis buffer (20 mM Tris, 500 mM NaCl, and 15% glycerol) containing 0.5 mg/mL of lysozyme (Sigma-Aldrich, Burlington, MA, USA) to soften the cell wall. Cell resuspension was lysed via ultrasonication on ice maintaining a low temperature against heat denaturation. The cell lysate centrifuged at 14,500 rpm for 30 min at 4 °C was collected and subsequently solubilized via incubation in 100 mL of Ni-NTA binding buffer (20 mM Tris, 500 mM NaCl, 5 mM imidazole, 6 M guanidine HCl, pH 8.0) for more than 12 h at room temperature with stirring. The centrifugation was repeated at 14,500 rpm for 30 min at 4 °C to remove the insoluble impurities. The supernatant was loaded onto a Ni-NTA column (Novagen Inc., Madison, WI, USA) previously charged with 50 mM NiSO_4_ and equilibrated in binding buffer. The column was washed with a washing buffer consisting of 16 mM imidazole to remove the non-specific bound proteins, and the bound proteins were eluted with 500 mM imidazole in elution buffer. The eluate containing fusion proteins was dialyzed against distilled deionized water at room temperature for 1 day using a 10,000 molecular weight cutoff (MWCO) membrane tubing (Repligen, Boston, MA, USA) to remove the denaturant and the salts. As the detergent was removed by dialysis, the denatured fusion proteins were restored to their native insoluble state and formed precipitates. The precipitates were collected and subsequently lyophilized.

The lyophilized fusion protein was dissolved in 70% formic acid (Sigma-Aldrich, Burlington, MA, USA) at a concentration of 5 mg/mL, and solid CNBr (Sigma-Aldrich, Burlington, MA, USA) at 100 mg/mL was added to chemically cleave the methionine residues present within the fusion protein selectively. The mixture was incubated in a dark room for 5 h at ambient temperature. After sufficient time for cleavage, the solution was diluted at least fivefold with distilled, deionized water and dialyzed against deionized water with a 1000 MWCO dialysis bag, followed by lyophilization. The purity of the purified protein at each step above was confirmed via 12% tris-tricine PAGE.

The hAPP-TM peptides were purified via semi-preparative reversed-phase HPLC on a Delta 600 HPLC system (Waters, Milford, MA, USA). The column was a Delta Pak C18 (7.8 mm × 300 mm, Waters) at a flow rate of 3 mL/min. Eluent A containing 0.1% trifluoroacetic acid (TFA) in 2% acetonitrile (ACN)/3% isopropanol/95% water and eluent B containing 0.1% trifluoroacetic acid (TFA) in 5% water/47% isopropanol/28% acetonitrile (ACN)/20% trifluoroethylene (TFE) were used. The protein mixture was dissolved in 25% hexafluoroisopropanol (HFIP)/75% methylene chloride (MC), and the insoluble part was removed by centrifugation (14,500 rpm, 4 °C, 30 min). The lyophilized peptide was dissolved in 1:3 HFIP/MC and placed in a bath sonicator for 30 min. At this stage, most of the KSI precipitates and aggregates were obtained. Only the supernatant except the precipitated KSI, was centrifuged for 30 min at 14,500 rpm at 4 °C. The soluble fraction was filtered through a 0.45-μm membrane filter, and then injected from an injection valve and a 10 mL sample loop. Chromatographic signals and associated UV spectra were acquired at 220 nm and 280 nm using a PDA detector. The identity and purity of purified hAPP-TM were established by 12% tris-tricine PAGE and mass spectrometry, followed by lyophilization.

### 2.2. Mass Spectrometry and CD Spectroscopy

The purified hAPP-TM peptide was analyzed by matrix-assisted laser desorption ionization time-of-flight (MALDI-TOF) mass spectrometry. The sample was prepared by dissolving the dried powders in 0.1% TFA/100% ACN, and 1 μL of the peptide solution was loaded on MALDI plate and completely dried. Then, 1 μL of CHCA matrix (α-cyano-4 hydroxylcinnamic acid) (Sigma-Aldrich, St. Louis, MO, USA) was loaded onto the peptide. The mass spectrum was obtained on a 4800 plus MALDI-TOF MS/TOF Analyzer; AB Sciex, Framingham, MA, USA). To improve the resolution and ionize the samples, the experiments were conducted using 355 nm Nd:YAG laser in reflector negative ion mode.

CD experiments were carried out using a Jasco J815 spectropolarimeter (Jasco, Easton, MD, USA) and 1 mm path-length quartz cuvette. The spectra were recorded between 190 and 260 nm with a data pitch of 0.2 nm, a bandwidth of 1 nm, a scan speed 50 nm/min, and a response time of 0.25 s. The peptides were prepared in 10 mM sodium phosphate buffer containing 20–100 mM dodecylphosphocholine (DPC) at pH 4.0. The data were averaged from five individual spectra. The measurement of the buffer without the peptide was subtracted to correct the baseline of the final spectra.

### 2.3. Solution-State NMR Spectroscopy

All solution-state NMR experiments were carried out using Bruker Avance III HD and Ascend^TM^ 400 MHz spectrometer (Bruker Biospin, Billerica, MA, USA) with z-gradient system. Micelle samples for solution-state experiments were prepared by dissolving 1 mg uniformly ^15^N-labeled hAPP-TM with 0.1 M DPC-d_38_ (Cambridge Isotope Laboratories, Andover, MA, USA) micelles in 400 μL H_2_O/D_2_O (90%/10%) at pH 4.0. The hAPP-TM powder samples were prepared at different concentrations (1.0 mM, 2.0 mM and 5.0 mM) to demonstrate multimer formation. Additionally, peptide samples for identification of zinc ion blockade effect were mixed with ZnCl_2_ (Junsei Chemical Co., Tokyo, Japan) at concentrations of 0 mM, 20.0 mM, 70.0 mM, 100.0 mM, respectively. The 2D ^1^H-^15^N heteronuclear single quantum coherence (HSQC) data were recorded at 313 K with 256 increments in F1 and 128 increments in F2 with 2048 complex points. Results were processed by TOPSPIN 4.0.6 (Bruker Biospin, Rheinstetten, Germany).

### 2.4. Solid-State NMR Spectroscopy

#### 2.4.1. ^15^N NMR Spectroscopy

To define the topology of hAPP-TM, the large bicelle (q = 3.2), which was fully mimicked to fluid bio-membrane, was adapted. The hAPP-TM samples for solid-state NMR spectroscopy were prepared with bicelles consisting of long- and short-chain ether-linked phospholipids (14-O-PC/6-O-PC). The concentration of uniformly ^15^N-labeled hAPP-TM peptide was prepared at about 3 mM (total volume 200 μL), which is considered to be a multimeric form in solution NMR experiment. Typically, the spectrum is observed in the upfield for transmembrane proteins and in the downfield for proteins located on the bicelle surface based on 120 ppm. Bicelles, which are perpendicular to the external magnetic field, were well aligned magnetically. This unflipped bicelle was preferred to define the structure of various peptides because of sample stability. The peptides in the bicelle placed under ^1^H-^15^N double-resonance, and treated with a solid-state NMR probe with a strip-shielded solenoidal coil, were measured via ^15^N 1D CP-MOIST (cross-polarization with mismatch-optimized IS transfer) and ^1^H-^15^N 2D SAMPI4 (selective averaging via magic sandwich pulses using π/4 flip pulses) experiments at 400 MHz Bruker AVANCE III spectrometer. Frequency pulse length of π/2 was 6 μs for ^1^H and ^15^N channel. We used 1024 complex points and 4096 scans. The contact time was 2 ms with a 20 Hz line broadening and a temperature of 298 K. All 1D solid NMR data were treated and displayed with Bruker Topspin 3.5 software. The 2D spectra were obtained with the SAMPI4 pulse sequence. The number of complex points was 1024 in the F2 dimension and the number of transients was 624 per spectrum acquisition in 42 increments to obtain 2D SAMPI4 spectra.

#### 2.4.2. Polarity Index Slant Angle (PISA) Wheel Pattern Analysis

The slant angle τ of the hAPP-TM was determined by fitting PISA wheel patterns to the 2D SAMPI4 spectra. The PISA wheel pattern of hAPP-TM was calculated using MATLAB & SIMUINK ver. R2010a (MathWorks, Inc., Natick, MA, USA). The fitted wheel projection provides secondary structure and topology information. PISA wheel pattern fitting was performed using ideal alpha helix Information. The chemical shift tensor was used with δ_11_ = 64, δ_22_ = 88 and δ_33_ = 222 for ^15^N and δ_11_ = 3, δ_22_ = 9 and δ_33_ = 17 for ^1^H. The standard order parameter S was 0.90 and the dihedral angle Φ was −57° and Ψ was −48° for hAPP-TM [33,34,35,36].

### 2.5. Preparation of Bicelle with hAPP-TM Peptide

Atomic force microscopy (AFM) was used to establish whether the final purified hAPP-TM peptide in bicelle was the ion channel. The bicelle sample was prepared as described for the use in solid-state NMR spectroscopy and was diluted 100-fold for AFM measurement. The experiment was conducted using Cypher VRS AFM (Asylum Research, Santa Barbara, CA, USA), and the AFM image was obtained in AC mode.

## 3. Results

### 3.1. Expression and Purification of hAPP-TM

**Figure 2:**
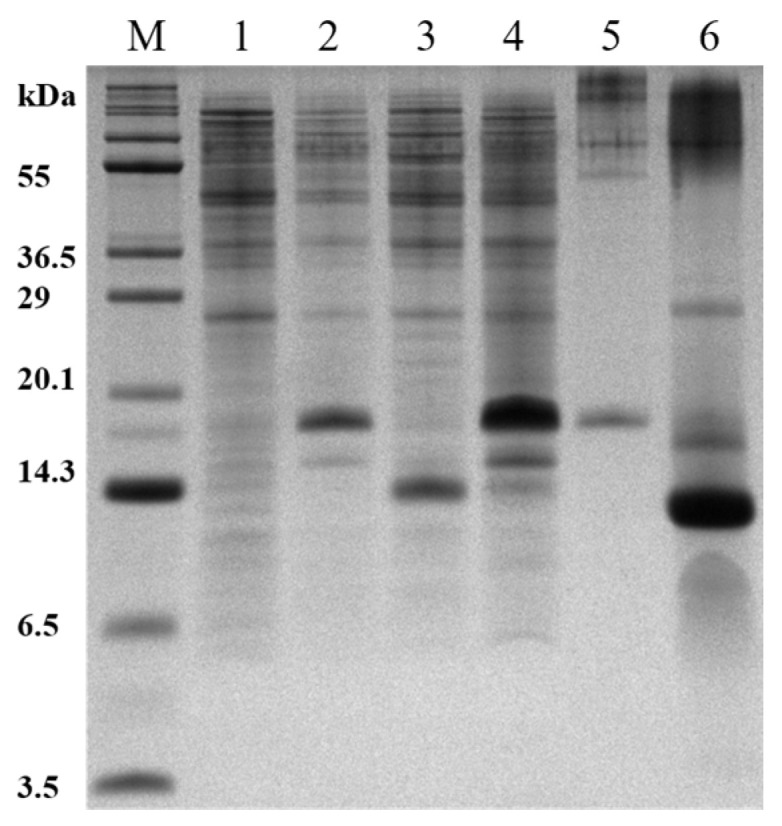
hAPP-TM expression and purification via 12% tris-tricine PAGE analysis. The different lanes indicate the different stages of purification: lane M, molecular weight marker; lane 1, whole cell before IPTG induction; lane 2, whole cell after IPTG Induction; lane 3, supernatant after lysis with lysozyme; lane 4, pellet containing fusion protein after lysis; lane 5, eluate of the KSI-fused protein from Ni-NTA affinity column; lane 6, fusion partner and target protein after chemical cleavage.

The hAPP-TM peptide was highly expressed and the purity at each experimental stage was evaluated via 12% SDS-PAGE (Figure 2). High-level expression of fusion protein was achieved by the addition of 1 mM IPTG at OD_600_ 0.5 (Figure 2, lane 2). KSI-fused protein was detected at approximately 18~19 kDa in the inclusion body of cells grown at 37 °C in M9 medium (Figure 2, lane 4) and purification was achieved using the insoluble fraction. Insoluble fusion protein was fully solubilized by unfolding with 6 M guanidine hydrochloride. His-tagged fused protein was efficiently purified via Ni-NTA affinity chromatography and then refolded by dialysis. We obtained a highly purified fusion protein (Figure 2, lane 5, approximately 18~19 kDa). KSI-fused protein was chemically cleaved with CNBr to release the hAPP-TM peptide (Figure 2, lane 6).

**Figure 3:**
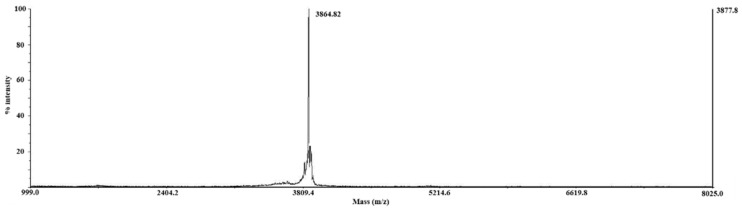
MALDI-TOF mass spectrum of uniformly ^15^N-labeled hAPP-TM acquired by HPLC purification. The measured molecular mass of monomeric hAPP-TM is 3864.82 Da, which is consistent with theoretical molecular weight (3870.34 Da) including C-terminal homoserine lactone form at C-terminus. Mass spectrum shows high purity and no degradation.

Following the chemical cleavage, the lyophilized fusion protein was purified via RP-HPLC using a C18 column. The elution profiles were monitored at 220 and 280 nm, and the fractions containing the protein were collected and analyzed via tris-tricine PAGE (Figure 3). Using RP-HPLC, two fractions were separated between 65 and 80 min, and confirmed via tris-tricine 12% gel as hAPP-TM peptides with two different forms: oxidized homoserine or homoserine lactone form. Oxidized homoserine form and homoserine lactone form are two forms that can appear in the chemical cleavage process using CNBr [37]. Taking advantage of the fact that the oxidized homoserine form was approximately 18 MW higher than that of the homoserine lactone form, it can be seen that the homoserine lactone form corresponds to lane 1 and the oxidized homoserine form corresponds to lane 2 in Figure 3.

In addition, the purified hAPP-TM peptide had a molecular weight of about 7–10 kDa in tris-tricine 12% gel, which could suggest dimer or a higher form of the hAPP-TM peptide under reducing conditions. The yield of the purified peptide was about 3–4 mg/L in the M9 minimal medium.

### 3.2. Structural Analysis

Purification and identification of purified peptide were performed via MALDI-TOF mass spectroscopy (Figure 3) [38]. As shown in Figure 3, MALDI-TOF MS revealed the complete purification of the peptide sample without impurities (theoretical molecular weight, 3870.34 Da), without additional contaminant peaks.

We analyzed the secondary structure of hAPP-TM peptide in the micelles via CD spectroscopy in a previous paper as a function of both the detergent concentration and pH value [31]. In this paper, for all DPC concentration micelles, double minima absorption (double dips) were observed at 208 and 222 nm, which are characteristic of the α-helical structure. This helical secondary structure (multimer or monomer) was very stable and remained the same in the range of DPC concentrations from 20 to 100 mM. Similar results were obtained when the multimer was analyzed by increasing the concentration, indicating that the multimer also contained an α-helical structure.

### 3.3. Solution-State NMR Spectroscopy

**Figure 4:**
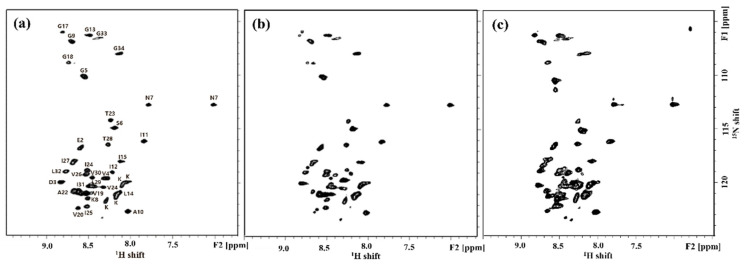
^1^H-^15^N 2D HSQC NMR spectra of hAPP-TM peptides with concentration (**a**) 1.0 mM, (**b**) 2.0 mM and (**c**) 5.0 mM.

^1^H-^15^N HSQC NMR experiments were carried out with ^15^N-labeled hAPP-TM (Figure 4). Each cross peak in the ^1^H-^15^N 2D HSQC spectrum provides a correlation between the amide proton and nitrogen in the peptide bond. In order to confirm the peptide multimers of hAPP-TM, an HSQC experiment was conducted with 1.0 mM, 2.0 mM and 5.0 mM peptide samples, and each of the cross peaks was assigned an ^1^H-^15^N HSQC spectrum and ^1^H-^15^N HMQC-NOESY spectrum (Data not shown).

As the protein concentration was increased from 1.0 mM to 5.0 mM (Figure 4a–c), the intensity of cross peaks was also increased, which might suggest formation of a homomultimer of the monomer [39,40,41]. In addition, it was found that the HSQC pattern did not change significantly even if the concentration of the peptide increased.

**Figure 5:**
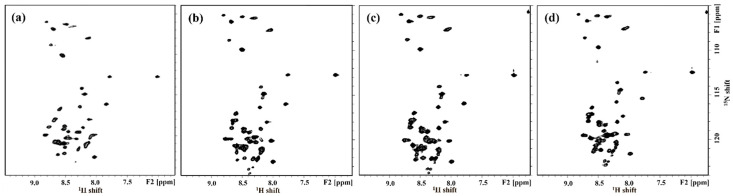
Uniformly ^15^N-labeled ^1^H-^15^N HSQC spectra demonstrate the inhibitory effect of zinc ions on hAPP-TM ion channel or pore. ^1^H-^15^N HSQC NMR spectra of samples in which uniformly ^15^N-labeled hAPP-TM peptides (1.0 mM) were mixed with ZnCl_2_ at concentrations of (**a**) 0.0 mM, (**b**) 20.0 mM, (**c**) 70.0 mM and (**d**) 100.0 mM.

In the micelle structure of a specific amount of hAPP-TM peptide, the ^1^H-^15^N HSQC spectrum was observed at various concentrations within the range of 0 to 100 mM of zinc chloride to investigate the relationship between the zinc ion concentration and the structural change (Figure 5).

**Figure 6:**
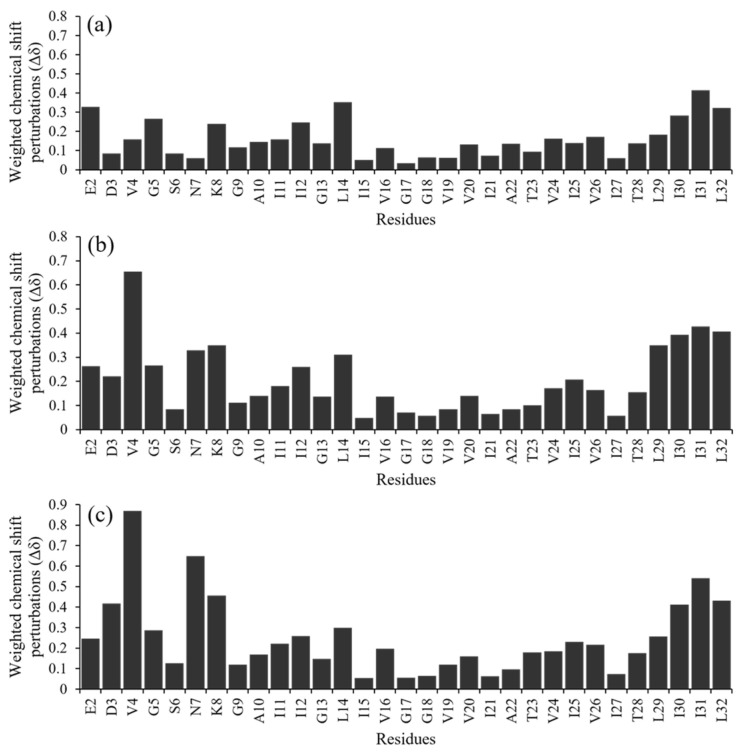
Chemical shift perturbation data of 1.0 mM hAPP-TM with different zinc ion concentration (**a**) 20 mM, (**b**) 70 mM and (**c**) 100 mM.

When comparing the sample spectrum without zinc chloride (Figure 5a) and the spectrum with zinc chloride (Figure 5b–d), the chemical shift of the residues at the end of the transmembrane domain occurred gradually, suggesting that the structure of hAPP-TM in the micelle might be changed gradually by the zinc ions. Increasing the concentration of zinc chloride to 70 mM altered the chemical shift of the peaks of additional residues in the transmembrane region (Figure 5c). The HSQC peak change when zinc ion was added in hAPP-TM was analyzed by chemical shift perturbation (CSP) (Figure 6). NMRFAM-SPARKY, CCPN analysis, and NMRbox programs were used for CSP analysis [42,43,44], and CSP was calculated using the following equation:

(1) $$CSP_{i}=\sqrt{{\left(∆\delta_{Hi}\right)}^{2}+\alpha {\left(∆\delta_{Ni}\right)}^{2}}$$

**Figure 7:**
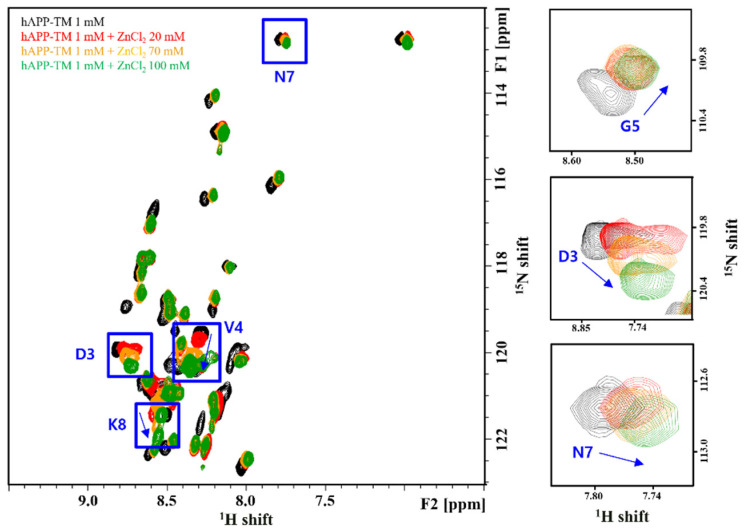
Overlay of 2D ^1^H-^15^N HSQC spectra of 1 mM hAPP-TM (black) with 20 mM ZnCl_2_ (red), 70 mM (orange) and 100 mM (green). The most prominent perturbed residues are marked with boxes.

When the zinc ion concentration was gradually increased, valine, the 4th residue on the N-terminal side, and the residues (N7, K8, D3, G5) around it were also affected, so that the chemical shift was significantly changed and I30~L32 of the C terminal was also significantly affected (Figure 7). In addition, as the peaks approached each other and clustered into one large peak, they could not be distinguished clearly. Considering these points, these findings suggest that as the concentration of zinc ions increased, the structure of hAPP-TM was lost gradually and exhibited a tendency to aggregate. A phenomenon in which some cross peaks overlapped each other was observed from a sample containing zinc ions at a concentration of 100 mM, suggesting the possibility of binding between zinc ions and specific moieties, followed by inhibition of multimer formation or blockade of the entry of calcium ions. A chemical shift occurred in some of the cross peaks, suggesting the possibility that the structure of hAPP-TM may be changed by binding of a zinc ion to a specific residue.

### 3.4. Solid-State NMR Spectroscopy

**Figure 8:**
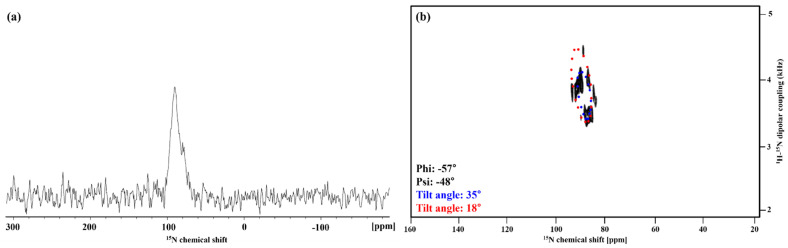
(**a**) The ^15^N 1D CP-MOIST spectra of hAPP-TM. The resonances for hAPP-TM ranged from 80 to 100 ppm in the spectrum suggesting that the peptides are parallel to the bilayer normal of ‘unflipped bicelle’, and thus the peptides are placed in a lipid membrane. (**b**) The ^15^N-^1^H 2D SAMPI4 spectrum of hAPP-TM.

In order to determine the inclination and the rotation angles of proteins in the actual biological membrane, solid-state NMR was used. A ^1^H-^15^N 1D CP experiment was performed by arranging hAPP-TM peptides on bicelle (q = 3.2) consisting of 14-O-PC and 6-O-PC. The unflipped bicelle aligned with the magnetic field lies perpendicular to the magnetic field and the bilayer normal. When the protein was attached to the surface of the unflipped bicelle under these conditions, a peak appeared in the upfield based on a ^15^N chemical shift of 120 ppm, and when the protein penetrated the membrane, a peak appeared in the downfield. The ^1^H-^15^N 1D CP spectrum of hAPP-TM revealed a peak located in the upfield of 120 ppm of ^15^N chemical shift, which suggests that hAPP-TM penetrated the bicelle membrane (Figure 8a).

The ^15^N-^1^H SAMPI4 solid-state NMR spectrum was obtained as a “wheel-like” pattern. Although this wheel pattern may appear simple, it provides important structural information. The peptide tilt in the biological membrane can be determined by fitting this spectrum via PISA wheel pattern analysis. The PISA wheel pattern of hAPP-TM based on the ^15^N-^1^H 2D SAMPI4 solid spectrum (Figure 8b) was obtained via optimization of the membrane tilt and the peptide of the alpha helix. As a result, it was expected that the dimer form of hAPP-TM would be located in the biological membrane, with a tilt of 18° and 32°, respectively. If the concentration was increased further, the tetramer form was expected in addition to the dimer form [22].

### 3.5. AFM Imaging of hAPP-TM in Bicelle

The structure of hAPP-TM peptide in the bicelle was established via AFM (data not shown). According to the obtained cross-section profile, the size of the bicelle with q = 3.2 showed a height of about 10 nm and a width of about 50 nm. However, the structure of hAPP-TM in the bicelle has yet to be clearly elucidated via additional experiments.

## 4. Conclusions

The structure of hAPP-TM, which is the main cause of AD, was investigated to determine the mechanisms of pathogenesis. Among the various hypotheses related to AD, the study was based on the hypothesis and simulation results suggesting that AG forms an ion channel in the biological membrane, and that neuronal cell death occurs following the entry of calcium ions through it. Bicelle experiments were performed in which the amino acid sequence of a membrane protein among the APPs was expressed and purified to identify the structure in the phospholipid. It was found that the purified hAPP-TM had an alpha-helical structure through CD spectroscopy. Through solution-state NMR spectroscopy, it can be seen, by the change in the peak pattern of the HSQC NMR spectrum, that multimers were formed due to the change in protein concentration. In addition, based on the HSQC spectra and CSP of hAPP-TM at various concentrations within the range of 10 to 100 mM of zinc chloride, we sought to elucidate the correlation between zinc ion concentration and structural change. Through the NMR CSP experiments, it was possible to find a significant change in CSP in the residue toward the N-terminus as the zinc concentration increased. This finding suggests the possibility that zinc ions bind to specific moieties and inhibit multimer formation or block the entry of calcium ions. Using solid-state NMR spectroscopy, it was confirmed that hAPP-TM carries a membrane protein inside the bicelle. The peptide topology was determined by calculating the inclination angle from the PISA wheel pattern analysis of the 2D ^1^H-^15^N SAMPI4 spectrum to indicate the angle of the hAPP-TM alpha helix in the normal phospholipid bilayer. The results suggested that hAPP-TM exists in a dimer form or tetramer of two different angles of helix, and that further multimer forms could be detected if the concentration was increased. Further experiments are necessary to establish the structure of the hAPP-TM peptide in the bicelle.

## Data Availability

Not applicable.
